# Depressive Disorder and Dermatological Autoimmune Diseases

**DOI:** 10.3390/jcm13113224

**Published:** 2024-05-30

**Authors:** Zuzanna Peła, Maria Gałecka, Agnieszka Murgrabia, Aneta Kondratowicz, Piotr Gałecki

**Affiliations:** Department of Adult Psychiatry, Medical University of Lodz, 92-213 Lodz, Poland; zuzanna.pela@gmail.com (Z.P.); maria.galecka@stud.umed.lodz.pl (M.G.); murgrabia.agnieszka@gmail.com (A.M.); aneta.kondratowicz@stud.umed.lodz.pl (A.K.)

**Keywords:** depression, depressive disorders, psoriasis, atopic dermatitis, alopecia areata, lichen planus, lupus, systemic scleroderma, vitiligo, Sjögren’s syndrome

## Abstract

Depressive disorders are a growing problem worldwide. They are also characterized by high comorbidity, including from the circle of dermatological diseases. Autoimmune diseases seem to be particularly correlated with depressive comorbidity, raising the question of their possible common pathomechanism. The PubMed database was searched, focusing on results published after 2016. A particular reciprocal correlation of depressive disorders with psoriasis, atopic dermatitis, alopecia areata, impetigo, lupus and systemic scleroderma was found. One possible explanation for the co-occurrence of the above diseases is that the inflammatory theory may be applicable to depression, the various elements of which also apply to autoimmune diseases.

## 1. Introduction

Mental illnesses are now becoming an increasingly serious social problem. In 2021, as much as 10% of all sick leave was issued precisely because of mental illness and behavioral disorders [1]. Among the aforementioned mental disorders, depression is a particularly significant problem, from which the WHO estimates that as much as 5% of the world’s adult population suffers [2].

According to the ICD-11 criteria, a depressive episode is characterized by a period of depressed mood or diminished interest in activities occurring most of the day or nearly every day during a period lasting at least two weeks accompanied by other symptoms such as difficulty concentrating, feelings of worthlessness or excessive or inappropriate guilt, hopelessness, recurrent thoughts of death or suicide, changes in appetite or sleep, psychomotor agitation or retardation, and reduced energy or fatigue. A depressive episode can be mild to severe, in which symptoms such as delusions or hallucinations may be present [3].

Depression is also a disease with high comorbidity. A higher prevalence of depressive disorders in migraine, chronic pain syndromes, malignancies, diabetes, cardiovascular disease, Parkinson’s disease and many others has been described [4].

According to a study, the risk of depression in patients with chronic comorbidities is about 50% compared with the general population, where such risk is about 8% [4]. Inflammatory dermatoses increase the population risk of depression to 10.1% and to 17.2% for anxiety disorders. Interestingly, almost 50% of people suffering from autoimmune disorders, including dermatologic disorders, also develop depressive or depression-like disorders, but without fully meeting the criteria of depression [5]. A study in the United States showed that dermatologic patients suffering from depression mainly exhibit symptoms such as anhedonia, fatigue, decreased appetite and decreased concentration [6]. It has been noted that psychiatric illnesses in dermatologic patients can exacerbate skin lesions, thus worsening the course of the underlying disease and overall quality of life [7]. Interestingly, however, a correlation in the opposite direction has also been observed, namely, the presence of depressive disorders predisposes a patient to a number of autoimmune diseases. 

A cohort study of more than 200,000 patients diagnosed with major depressive disorder in Taiwan showed that these patients had a statistically significant risk of autoimmune comorbidities, with an adjusted risk ratio of 10.41%. Patients with major depressive disorder have a significantly higher risk of developing psoriasis, lichen planus, alopecia areata, limited scleroderma, autoimmune blistering diseases, purulent eyelid inflammation, acquired vitiligo, lupus erythematosus, systemic scleroderma, Sjögren’s syndrome and dermatomyositis [8]. The most important of these will be reviewed below.

## 2. Materials and Methods

**Figure 1:**
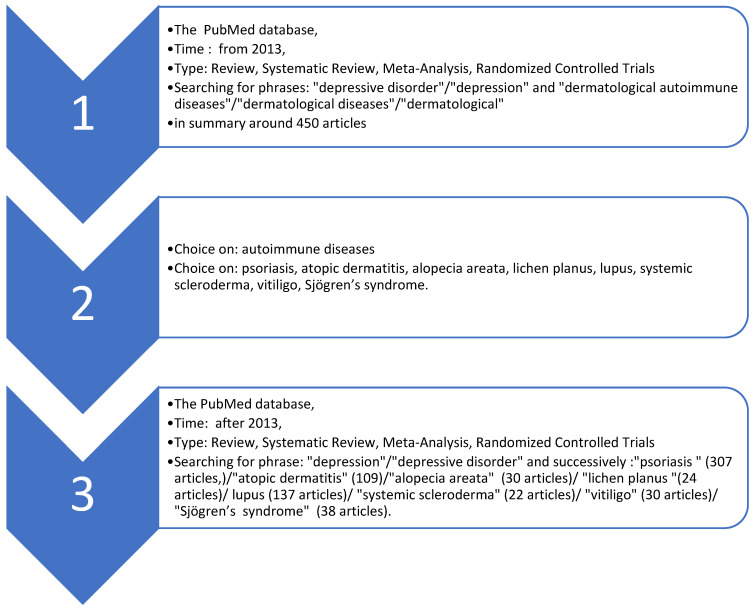
Decision-making schema.

First, the PubMed database was chosen as one of the most well-known for its reliability. Meta-analyses, randomized controlled trials, reviews and systematic reviews were included in the review. Clinical trials were excluded. The year 2013 was chosen as the start of a 10-year period relative to the date of the study’s creation and also as the period containing current reports (Figure 1).

Therefore, the focus was on articles published after 2013 on recent reports linking the issues of dermatological diseases and depression. At this stage, it was a general review to identify the available types of studies and the evidence in a given field. The analysis aimed to determine which dermatological diseases had the most frequent correlation with depressive disorders. An initial search of the PubMed database from 2013 using the phrases “depressive disorder”/“depression” and “dermatological autoimmune diseases”/“dermatological diseases”/“dermatological” was conducted, yielding a total of about 450 articles in summary. After analysis of the material, autoimmune diseases were singled out as the most common comorbidities with depressive disorders. 

Eight of them were selected as the most common in studies on the co-occurrence of depressive disorders and dermatological diseases. In addition, these diseases also have a high prevalence compared with other autoimmune depressive diseases and are observed in daily inpatient and outpatient practice. The observed social impact (for example, the need for hospitalization and sick leave) associated with the disease was also taken into account. 

The following diseases were singled out as particularly relevant to depressive disorders: psoriasis, atopic dermatitis, alopecia areata, lichen planus, lupus, systemic scleroderma, acquired vitiligo and Sjögren’s syndrome.

The PubMed database was then reviewed again from 2013 by searching for the phrases “depression”/“depressive disorder” and the following disease entities. The following numbers of articles were obtained: psoriasis, 307 articles; atopic dermatitis, 109; alopecia areata, 30; lichen planus, 24; lupus, 137; systemic scleroderma, 22; vitiligo, 30; Sjögren’s syndrome, 38. 

Meta-analyses, randomized controlled trials, reviews and systematic reviews were included in the review. Clinical trials were excluded. Articles that included the selected phrase only as a mention and did not refer to an explanation of the selected diseases’ comorbidities, treatment, possible pathomechanism or any other connected thing were not taken into account when analyzing them subsequently.

The purpose of this part of the study was to identify some common factors between autoimmune dermatological diseases and depressive diseases, and to clarify the concept in a given field. 

The most important of the articles were then briefly analyzed. In line with the results indicating the inflammatory pathway present in depression and autoimmune dermatological diseases, an attempt was made to describe the possible relationships in accordance with new reports regarding the theory of depressive disorders.

A descriptive review of the results of the literature search was conducted. This article is based on previously conducted studies and does not include any participant or animal studies conducted by any of the authors.

## 3. Results

### 3.1. Depressive Disorders and Psoriasis

Psoriasis is a common chronic skin disease (characterized by areas of abnormal skin), affecting 0.6–4.8% of the population of all ages and worldwide [9]. It is a consequence of genetic susceptibility, and environmental factors such as streptococcal infection, stress, smoking, obesity and alcohol consumption. Psoriasis cannot currently be cured, but treatment should seek to minimize physical and psychological damage by treating patients early in the process of the disease, identifying and preventing associated comorbidities, modifying lifestyles and using a personalized approach to treatment [10].

Psoriasis, like depression, is characterized by high comorbidity [11]. Patients have been found to have a higher risk of metabolic disease, cardiovascular disease and mental illness than the general population, which is associated with general inflammation [12,13]. As indicated by an analysis of studies by B. Ferreira et al., 24% to 90% of patients with psoriasis also suffer from psychiatric disorders [14].

There is evidence of a higher risk of depressive symptoms, other mood disorders, personality disorders (17.5%), sleep disorders (56.6%), psychosis (up to 35%) or sexual dysfunction (20.9%) [15]. According to the study, 82.8% of psoriasis cases also co-occur with symptoms of anxiety, and 33% of patients are diagnosed with generalized anxiety disorder. Moreover, 68% of patients with psoriasis are addicted to nicotine, and up to 32% may manifest symptoms of alcohol dependence [14]. These patients also show twice the risk of suicidal behavior [11]. 

For the co-occurrence of many of the abovementioned psychiatric disorders with psoriasis, there is no clear explanation. 

Depression, however, stands out because of the increasing amount of research devoted to the inflammatory pathway, explained in more detail later in this article. An inflammatory pathomechanism has been described in depression and in psoriasis, as well as in the other autoimmune diseases mentioned in this article. In depression, it has been noted that abnormalities of the HPA axis affect 50–75% of patients. Moreover, patients diagnosed with depressive disorders have been observed to have increased central and peripheral levels of a number of pro-inflammatory cytokines, mainly tumor necrosis factor α (TNF-α) and interleukins (ILs) [16]. 

Psoriasis, on the other hand, is an autoimmune disease in which an increase in pro-inflammatory cytokines has also been described. In psoriasis, premature maturation of keratinocytes can be observed, which is induced by an inflammatory cascade in the dermis involving dendritic cells, macrophages and T cells. These immune cells subsequently secrete inflammatory cytokines such as interleukins, or tumor necrosis factor [17,18,19]. 

Depressive symptoms can occur in up to 68% of patients with psoriasis [14]. It is now suggested that the relationship between psoriasis and depression is bidirectional, with a common pathomechanism of activation of the inflammatory system playing an important role. Pro-inflammatory cytokines, such as tumor necrosis factor (TNF), are involved in the pathogenesis of both psoriasis and depression [20]. These levels directly correlate with the severity of psoriasis. Similarly, it has been discussed that patients with severe depression have higher peripheral TNF levels, which also correlate with the disease’s severity [21]. 

However, it is worth mentioning briefly that the social impact of psoriasis and social stigma also play a role in the co-occurrence of both disease entities, contributing to an increased risk of depression and anxiety [22]. Psoriasis can affect physical appearance, which can lead to stigma, limitations in social interaction and isolation [23]. Poor quality of life appears to be correlated with higher rates of depression, anxiety and even suicidal thoughts [22]. It has also been described that exacerbation of psoriasis occurs after episodes of psychosocial stress [24].

But, to return to the main topic focusing on the issue of the inflammatory pathway, it is worth citing other studies. Other studies also indicated higher levels of sIL6R in both depression and inflammation. sIL6R can cross the blood–brain barrier, causing an increase in Il-6 in the CNS [21]. Subsequently, the expression of indoleamine dioxygenase (IDO) increases, which decreases tryptophan levels and promotes the production of its catabolites, such as kynurenine and quinolinic acid. As described in a study by Harden et al., the expression of L-kynureinase (an enzyme of tryptophan metabolism) is positively correlated with the incidence of psoriasis and disease severity. A growing number of studies have also indicate elevated levels of kynurenine, quinolinic acid and the aforementioned Il-6 in depression [25].

The levels of the increased cytokines in depression and psoriasis are displayed in Table 1.

According to recent studies, elevated levels of tumor necrosis factor alpha; interleukins 1, 2, 10, 1 beta, 6 and 8; prostaglandin E2 and interferon gamma are suggested to be present in both psoriasis and depression [26]. 

In a small Danish study, the role of serotonin transporter (SERT) was investigated. A positive correlation between the expression of SERT and the severity of psoriasis was found, which suggests that the serotonergic system, a well-known neurohormonal mediator in depression, might also have a role in the pathogenesis of psoriasis [27]. In the discussion concerning the connection of psoriasis with depression, other neurotransmitters have also been mentioned. In healthy tissue, mast cells are responsible for the skin’s response to stress and neurogenic inflammatory reactions. Some neuropeptides related to the response to stress, including CRH, substance P (SP) and CGRP, act in a pro-secretory manner on the mast cells. Another interesting theory, apart from inflammation, is that sunlight is not involved in both cases. The insufficiency of daylight is considered an important factor of depression occurring at higher geographical altitudes. Moreover, one of the commonly used therapies in psoriasis is light therapy with PUVA or NB-UVB [21].

Many patients diagnosed with psoriasis suffer also from sleep disorders. The cross-section of studies indicated a wide variety of prevalence from 0.05% to 77.1%, which was described by a study from 2022 by Halioua et al. [9]. During the studies, the researchers used interviews and questionnaires, e.g., the Pittsburgh Sleep Quality Index (PSQI). Five case–control studies showed significantly higher scores of PSQI in patients suffering from psoriasis than in the control group. One case–control study indicated a 4.3-fold higher risk of sleeplessness in patients with psoriasis. Some data suggested a positive correlation between the severity of psoriasis, assessed by the scale called the Psoriasis Area and Severity Index (PASI), and the risk of developing sleep disorders. A positive correlation was also demonstrated between comorbid psoriatic arthritis and the risk of sleep disorders. This might come also from the higher PASI scores in people suffering from psoriasis arthritis. One of the most severe sleep disorders which are comorbid with psoriasis is obstructive sleep apnea. Interestingly, CPAP (Continuous Positive Airway Pressure) therapy might have a positive influence on psoriatic skin lesions and cause a decrease in TNF-alpha or Il-6 [9]. 

As mentioned, the links between psoriasis and depression have been known for many years. For example, a study describing the psychosomatic aspects of depression was published as early as 1990 [28], with the link mentioned much earlier. Despite this, specific guidelines for the treatment of depression in psoriasis have not been developed. An episode of depression can trigger an exacerbation of skin lesions and, conversely, its symptoms worsen in psoriasis [29].

In depression, the immune system is in a state of heightened activity, and studies describing the effects of antidepressants in depression point to their immunomodulatory and anti-inflammatory effects. The question arises whether the use of antidepressants is helpful in the treatment of psoriasis. There has not been much research in this area. One study suggested a positive effect of monoaminergic antidepressants on the development of psoriasis [30]. Another large cohort study found that people with psoriasis who took serotonin reuptake inhibitor antidepressants had a significantly lower risk of needing systemic psoriasis treatment [31]. 

An agent that is particularly correlated with cognitive impairment in depressive disorders and psoriasis is tumor necrosis factor (TNF)-alpha. Serotonin reuptake inhibitors reduce levels of IL-6 and tumor necrosis factor [32]. 

Given the functional integration between the immune and nervous systems, and the prevalence of the co-occurrence of depression and psoriasis, the medical community, on the basis of previous research and the experience of psychiatrists and dermatologists, should develop guidelines for the management of the co-occurrence of both diseases. This will allow a comprehensive approach to the patient. 

### 3.2. Atopic Dermatitis and Depressive Disorder

Atopic dermatitis is a chronic, inflammatory disease in which the epidermal barrier is damaged, leading to inflammatory lesions of the skin. The epidermal barrier is one of body’s natural barriers that protects it against harmful external factors. This disease is characterized by numerous skin lesions, severe itching (caused by the release of histamine) and excessive skin dryness (related to excessive water loss) [33]. Most patients suffering from atopic dermatitis struggle with chronic itching, sleep disorders and also social stigmatization. All of these are caused by numerous visible skin lesions [34]. The abovementioned problems significantly reduce the quality of life, as social relationships usually deteriorate and are impoverished. It is not uncommon for the affected patients to experience social isolation and a significant reduction in self-esteem [35]. Studies have shown that patients with atopic dermatitis have a higher risk of developing depression and anxiety disorders. Some groups of patients have an increased risk of suicide attempts, which may result in a higher percentage of completed suicides [36]. 

Chronic inflammation is characteristic of atopic dermatitis, primarily caused by increased levels of pro- inflammatory cytokines such as IL-6 or TNF-alpha. Elevated levels of these substances also occur in depressive disorders. In stressful situations, pro-inflammatory cytokines penetrate the blood–brain barrier, provoking the disruption of the central nervous system. Increased levels of TNF-alpha lead to a faster breakdown of neurotransmitters such as serotonin. IL-6 leads to increased levels of ACTH and cortisol in the blood. This results in disorders in the functioning of the HTPA axis [37]. As can be seen, the underlying mechanisms of atopic dermatitis share similarities with those of depression. 

The cause of chronic fatigue in patients is sleep disturbance. The main problems are usually a shorter sleep duration, numerous awakenings during the night and difficulty falling asleep. These abnormalities also result in repeated mood disorders and increase the risk of depression [34].

Studies conducted on the Danish population showed that patients suffering from atopic dermatitis were treated with antidepressants and anxiolytics much more often than patients without atopic dermatitis. It was also found that affected women are twice as likely to suffer from depression than affected men [38].

Dupilumab is a human monoclonal antibody directed against interleukins 4 and 13. Those interleukins are secreted by Th2 cells and they play a major role in the pathogenesis of asthma, allergic rhinitis and atopic dermatitis [39]. 

This drug, administered by subcutaneous injection, is used to treat atopic dermatitis. It has an advantage over classic immunosuppressive drugs, such as methotrexate or cyclosporine, which have a higher risk of side effects [40].

Interestingly, it has been shown that dupilumab alleviates not only the symptoms of atopic dermatitis, such as skin dryness and itching, but also reduces depressive symptoms and mood disorders. The quality of sleep in patients also improves. This phenomenon is caused by increased self-confidence and reduced levels of social isolation in patients whose skin lesions become calmer. Less severe itching allows them to return to regular work or study. It has a positive effect on the development of social relationships [41]. 

It is also worth mentioning that according to recent studies, mirtazapine, one of the antidepressants, is used to relieve the symptoms of both psoriasis and atopic dermatitis [42].

### 3.3. Alopecia Areata and Depressive Disorders

In alopecia areata there is a loss of hair from some or all areas of the body. Patients with alopecia areata (AA) have a higher risk of being diagnosed with anxiety and/or depression and experiencing impaired quality of life [43,44]. In a large meta-analysis, it was found that between 7% and 17% of AA patients suffered from depressive or anxiety disorders requiring psychiatric care, including specific medications. In addition, more than one-third of the patients had symptoms that are warning signs that require monitoring because they can develop into disorders [45]. An emerging explanation for the observed correlation is the social stigma associated with the impact of alopecia areata on the appearance, but it is worth noting that another type of alopecia—androgenetic alopecia—was correlated with decreased quality of life but did not correlate with depressive symptoms [46]. 

It is still worth noting that studies have shown a reciprocal correlation of comorbidity between alopecia and depression in both directions. Not only were patients with alopecia areata found to have an increase in depressive disorders, but also vice versa. In the aforementioned Taiwanese cohort study of more than 200,000 patients diagnosed with major depressive disorder, a statistically significant risk of comorbidity with alopecia areata was found in these patients [8]. In a retrospective cohort study conducted between 1986 and 2012 in outpatient clinics in the UK, major depressive disorder was shown to increase the risk of later developing AA by 90%, while antidepressants showed a protective effect on the risk of AA. In the other direction, similarly, AA increases the risk of later developing major depressive disorder by 34% [47]. In contrast, another study in Taiwan found a bidirectional association between AA and MDD (major depressive disorder) among probands and healthy siblings, suggesting common family mechanisms underlying AA and MDD [48].

With this correlation, the question of the pathogenesis of depressive disorders and lichen planus definitely arises. CD8 + NKG2D + T lymphocytes dominate the pathogenesis of hair follicles, but the specific mechanisms causing hair loss are not fully understood. A study was conducted that analyzed the cytokines in the plasma of patients with alopecia areata. It found that people with alopecia areata had increased plasma levels of the Type 2 cytokines IL-33, IL-31 and IL-17E (IL-25), in addition to the Type 17 cytokines IL-17A, IL-21, IL-23 and IL-17F. Alopecia areata is also connected with changes in levels of interleukin (IL)-6, tumor necrosis factor-α, IL-1β and Type 17 cytokines. In doing so, levels of IL-17E and IL-22 are correlated with the co-occurrence of depressive disorders [49].

Another study described that the active disease stage of alopecia areata was connected with a decrease in the number of circulating CD4, CD8 and NK T cells and an increase in the CD4/CD8 T cell ratio; however, the levels of these cells were higher with increasing disease duration. This shows that immune mechanisms are involved in the pathogenesis of AA [50]. 

Another study found that in alopecia areata, various immune cell lineages, including plasmocytoid dendritic cells, NK cells and T cells, along with key molecules such as interferon-γ, interleukin-15, MICA and NKG2D, contribute to the autoimmune process [51].

### 3.4. Lichen Planus and Depressive Disorders

Lichen planus is a chronic autoimmune inflammatory disease that comes in several different forms. 

In a large study conducted in the United States, lichen planus was shown to be significantly correlated with the comorbidities of depression (OR, 1.36; 95% CI, 1.20–1.56, *p* < 0.001) and anxiety (OR, 1.48; 95% CI, 1.30–1.68, *p* < 0.001) [52], which was indicated in other studies [53,54,55]; this affects the quality of life of patients with lichen planus. Many studies specifically refer to the co-occurrence of depression and anxiety in patients with the oral form of lichen planus [56,57,58,59]. The correlation of oral lichen planus with depression, anxiety and stress was also discussed by a meta-analysis last year [60].

There was a study that measured the expression of serotonin in skin biopsy specimens, finding significantly higher scores in patients with oral lichen planus [61]. A study was also published, in which the search for factors common to depressive disorders and lichen planus highlighted two genes (NCAM1 and CD4) identified as common to the pathomechanism of both disorders [62]. Patients with the oral form of lichen planus were also found to have higher salivary alpha-amylase levels in those with depressive disorders, which may be an interesting further line of research into the risk markers for the development of depressive disorders [63]. However, the most interesting study from the point of view of inflammatory theory and depressive disorders is the one that found increased levels of stress, mast cells and salivary nitric oxide in patients with depressive disorders and lichen planus [64].

### 3.5. Lupus and Depressive Disorder

Lupus is an autoimmune disease with many symptoms, including dermatological symptoms, for example, a red rash, which is most commonly seen on the face; mouth ulcers and hair loss. It is estimated that between 11% and as many as 71% of lupus patients also meet the criteria for depressive disorders [65].

In general, it is recognized that anxiety and depression are the most commonly diagnosed psychiatric symptoms in lupus, with anxiety correlated most often with myalgia, and depressive disorders correlated with unmarried status, smoking, damage to specific organs and the severity of SLE; this study also found that depression is as common in lupus as in other inflammatory rheumatic diseases [66]. Interestingly, another study found a correlation of depressive disorders with age, alopecia, level of inflammation and kidney involvement [67].

The factors underlying the common mechanism of depressive disorders with lupus erythematosus remain largely unknown.

One study set out to test the correlations among depressive disorders, anxiety and disease activity. It was found that the comorbidity of depression means an increase in the disease’s activity, while anxiety was correlated with levels of anti-PO antibodies. Interestingly, both depression and anxiety correlated with the co-occurrence of proteinuria. HAMA and HAMD scores had a strong positive correlation and were independent risk factors for each other. The correlation between depression and disease activity in SLE may reveal underlying damage to the central nervous system in SLE. The role of anti-PO antibodies in emotionally disturbed SLE patients requires further study [68].

It has also been hypothesized that the common pathogenesis of lupus and emotional disorders should be sought in the damage to the nervous system following the presence of pro-inflammatory cytokines, again leading to the inflammatory theory of depression [68,69].

It is still worth mentioning that just 1 year earlier than the accepted study period for this article, a large systematic review was published, which showed that depressive disorders and cognitive impairment are more common in patients with lupus. Again, inflammatory factors, activation of the microglia, hypocoagulation, oxidative stress, mitochondrial dysfunction and blood–brain barrier disruption have been postulated as possible pathomechanisms [70].

However, regardless of the pathomechanism, it should be emphasized that the presence of depressive disorders adversely affects the course and prognosis of lupus [71]. Studies have emphasized the major role of appropriate pain management as a factor that provides an appropriate advantage against the development of depressive disorders [65]. However, it was suggested in the 2010 recommendations that, in general, depressive disorders in lupus should be treated first as a separate disease entity [72].

### 3.6. Sclerosis and Depressive Disorder

Systemic sclerosis is a chronic disease characterized by vasculopathies, inflammation and fibrosis of the skin and organs. Its symptoms include interstitial lung disease, pulmonary hypertension, digital ulcers, gastrointestinal symptoms, skin fibrosis and joint contractures [73]. This disease occurs in 0.05% of the population [74], and 73.3% of scleroderma patients experience sleep disturbances. As the study suggested, this is correlated with the involvement of the esophagus, the severity of the disease and depressive states [75]. Another study conducted over an 8-year period, involving 316 patients who met the ACR/EULAR 2013 criteria, assessed their mental condition according to the HADS (Hospital Anxiety and Depression Scale). It showed the occurrence of anxiety disorders (32.2%), depression (25.9%) and depressive-anxiety disorders (18.5%) in these patients. Moreover, 49% of patients experienced states of emotional stress [73]. Similar results were also shown in another study focusing on anxiety and depressive disorders in patients with rheumatological diseases. Patients with systemic sclerosis, also assessed with the HADS scale, had depressive disorders (35%) and anxiety disorders (41%) [76]. One of the largest studies on this topic examined 15,141 patients aged 63.32 ± 18.06 years. In this case, the prevalence of depression in patients with scleroderma was 16.2% and was significantly higher than in the control group (10.9%). This proportion was even higher in females and in patients with low socioeconomic status. The study did not demonstrate an association between systemic sclerosis-specific autoantibodies (i.e., anti-centromere antibodies (ACA), anti-Scl-70, anti-polymerase III or anti-RNP) and the risk of depression in patients with scleroderma. It also did not have a statistically significant impact on survival in these patients [74].

### 3.7. Vitiligo and Depressive Disorders

Vitiligo is an autoimmune disease that causes chronic skin depigmentation. About 1% of the world’s population suffers from it [77].

Several studies have shown the co-occurrence of depressive disorders and vitiligo [78,79,80,81]. Women, those with either visible or genital lesions, those under 30 years of age and those with lesions covering a larger portion of the body were particularly vulnerable to the most common comorbid psychiatric disorder [81].

However, it has not been established exactly what pathomechanism is associated with the co-occurrence of depressive disorders and vitiligo. Further research is indicated to answer the question of whether the depressive disorders are mainly related to the impact of vitiligo on self-esteem, social status, etc., or whether there is also a common pathomechanism here.

An interesting pathway in this regard seems to be the altered expression of the gene encoding human β-defensin 1 (a small protein, part of the immune system and the inflammatory reaction), which has been linked to depressive disorders, vitiligo and lupus, among other things [82].

### 3.8. Sjögren’s Syndrome and Depressive Disorders

Sjögren’s syndrome is a chronic autoimmune disease involving damage to the exocrine glands, primarily the salivary and lacrimal glands. A meta-analysis showed an increased prevalence of depressive disorders in patients with Sjögren’s syndrome [83]. The pathomechanism remains unclear, but, as in previous diseases, the increased activity of pro-inflammatory cytokines has also been suggested [84].

## 4. Discussion

In view of such a high correlation of comorbidities, the question arises about the etiology of depressive disorders and autoimmune diseases. At this point, however, it is necessary to briefly mention once again the inflammatory theory of depression, which has enabled further research into the relationship between depression and autoimmune diseases. It assumes that overactivity of the hypothalamic–pituitary–adrenal axis and dysregulation of the immune system are the source of abnormalities in the kynurenine pathway. This, in turn, translates into a reduced amount of tryptophan necessary for the production of serotonin. The observation of the co-occurrence of depressive disorders with a number of indicators characteristic of the chronic inflammatory process has contributed significantly to the inflammatory theory of depression. It has been noted that abnormalities of the HPA axis affect 50–75% of patients diagnosed with major depression. Moreover, patients diagnosed with depressive disorders have been observed to have increased central and peripheral levels of a number of pro-inflammatory cytokines, mainly tumor necrosis factor α (TNF-α) and interleukins (ILs) [85]. This theory is now finding increasing confirmation. A relationship of a bidirectional link between inflammation and depressive disorders has also been described [86,87]. A recent meta-analysis also indicated the association of depression with inflammation, present both at the onset of depressive disorders and in the future, in children and young adults [88].

There remains the question of the neurodevelopmental theory of depression, which is an extension of the inflammatory theory of depression. According to this, chronic activation of the pro-inflammatory process that occurs in depressive disorders in pregnant women leads to increased vulnerability to depression in their children [16]. Moreover, it is not only depressive disorders that lead to increased levels of pro-inflammatory cytokines, but also a number of diseases of civilization, as well as autoimmune diseases. Adults with maternal activation of the pro-inflammatory cytokine system during pregnancy have lower susceptibility to stress, higher levels of anxiety, a greater risk of developing an avoidant personality and, finally, a greater susceptibility to depressive disorders and autoimmune diseases [89]. Indeed, an additional puzzle is the theory that prenatal cytotoxicity through excessive activation of the inflammatory system causes the abnormal differentiation of CD4 + cells in adulthood, which predisposes an individual to depressive and autoimmune disorders; conversely, autoimmune disorders in a pregnant woman may contribute to increased susceptibility to depressive disorders in the offspring [90].

The links shown in the article between depressive disorders and autoimmune dermatological diseases also seem to support the theories briefly cited above.

With the consistent assumption that these disorders can induce each other, including in subsequent generations, further research into their common pathomechanism and treatment seems particularly needed.

## 5. Limitations

The study is an overview and contains some limitations. The first limitation is the focus on the PubMed electronic database; other databases were not searched. Therefore, additional relevant studies may have been missed. Additionally, phrases relating to depression/depressive disorders and dermatological diseases or individual diseases were searched for. In this way, studies that did not contain the phrases listed in the methods may have been omitted. Thirdly, the selected time period from 2013 may be insufficient to fully describe the relationship between depressive disorders and dermatological diseases.

## 6. Conclusions

With autoimmune dermatological diseases such as psoriasis, atopic dermatitis, alopecia areata, lichen planus, lupus, systemic scleroderma, vitiligo and Sjögren’s syndrome, depressive disorders co-occur significantly more often.

The relationship defining the co-occurrence of depressive disorders and autoimmune dermatological diseases is bidirectional.

Depressive disorders and autoimmune dermatological diseases appear to share a common pathomechanism based on activation of the inflammatory pathway, which may explain their co-occurrence.

There are reports of a positive effect of antidepressants in autoimmune dermatological disorders.

Further research in this area is indicated.

## Figures and Tables

**Table 1:** Increased levels of pro-inflammatory cytokines in psoriasis and depression.

Psoriasis	Depression
TNF-α,	TNF-α,
IL-12,	IL-1,
IL-17,	IL-1β,
IL-23,	IL-2,
INF-γ.	IL-6,
	IL-8,
	IL-17,
	IL-21,
	IL-23,
	CRP,
	TGF-β.
